# TORC1 Regulates Thermotolerance via Modulating Metabolic Rate and Antioxidant Capacity in Scallop *Argopecten irradians irradians*

**DOI:** 10.3390/antiox13111359

**Published:** 2024-11-06

**Authors:** Longfei Chu, Ancheng Liu, Jiaxi Chang, Junhao Zhang, Xiujiang Hou, Xinghai Zhu, Qiang Xing, Zhenmin Bao

**Affiliations:** 1 MOE Key Laboratory of Marine Genetics and Breeding, College of Marine Life Sciences, Ocean University of China, 5 Yushan Road, Qingdao 266003, China; chulongfei@stu.ouc.edu.cn (L.C.); liuancheng@stu.ouc.edu.cn (A.L.); changjiaxi@stu.ouc.edu.cn (J.C.); zjh6667@stu.ouc.edu.cn (J.Z.); houxiujiang@stu.ouc.edu.cn (X.H.); xhzhu@ouc.edu.cn (X.Z.); zmbao@ouc.edu.cn (Z.B.); 2 Laboratory for Marine Fisheries Science and Food Production Processes, Qingdao Marine Science and Technology Center, Qingdao 266237, China

**Keywords:** *Argopecten irradians irradians*, TORC1, genome-wide identification, expression regulation, functional allocation, thermotolerance

## Abstract

Target of rapamycin complex 1 (TORC1) is a key regulator of metabolism in eukaryotes across multiple pathways. Although TORC1 has been extensively studied in vertebrates and some invertebrates, research on this complex in scallops is limited. In this study, we identified the genes encoding TORC1 complex subunits in the scallop *Argopecten irradians irradians* through genome-wide in silico scanning. Five genes, including *TOR*, *RAPTOR*, *LST8*, *DEPTOR*, and *PRAS40*, that encode the subunits of TORC1 complex were identified in the bay scallop. We then conducted structural characterization and phylogenetic analysis of the *A. i. irradians* TORC1 (AiTORC1) subunits to determine their structural features and evolutionary relationships. Next, we analyzed the spatiotemporal expressions of AiTORC1-coding genes during various embryo/larvae developmental stages and across different tissues in healthy adult scallops. The results revealed stage- and tissue-specific expression patterns, suggesting diverse roles in development and growth. Furthermore, the regulation of AiTORC1-coding genes was examined in temperature-sensitive tissues (the mantle, gill, hemocyte, and heart) of bay scallops exposed to high-temperature (32 °C) stress over different durations (0 h, 6 h, 12 h, 24 h, 3 d, 6 d, and 10 d). The expression of AiTORC1-coding genes was predominantly suppressed in the hemocyte but was generally activated in the mantle, gill, and heart, indicating a tissue-specific response to heat stress. Finally, functional validation was performed using the TOR inhibitor rapamycin to suppress AiTORC1, leading to an enhanced catabolism, a decreased antioxidant capacity, and a significant reduction in thermotolerance in bay scallops. Collectively, this study elucidates the presence, structural features, evolutional relationships, expression profiles, and roles in antioxidant capacity and metabolism regulation of AiTORC1 in the bay scallop, providing a preliminary understanding of its versatile functions in response to high-temperature challenges in marine mollusks.

## 1. Introduction

Since the Industrial Revolution, global temperatures have consistently risen due to the greenhouse effect, which has significantly intensified in recent years. Notably, over 90% of the excess heat from this effect has been absorbed by the oceans, owing to their remarkable heat retention capacity [1]. Concomitantly, the increase in oceanic heat has led to more frequent and prolonged marine heatwaves, resulting in unusually high sea surface temperatures (SST) across various global oceanic regions [2,3,4,5]. The escalating sea temperature can stimulate various metabolic processes in marine organisms, increase oxygen consumption, lead to the production of reactive oxygen species (ROS), and cause oxidative stress [6]. And it has adversely affected marine organisms across multiple dimensions encompassing growth, development, population dynamics, migration patterns, community structure, and ecosystem stability. For instance, elevated water temperatures have reduced the lipid content in the gonads and oocytes of *Argopecten purpuratus* (Lamarck 1819), affecting larval viability depending on egg-derived nutrients [7,8]. Increased temperatures have also exacerbated metabolic constraints on fish by altering their growth trajectories [9] and have also been linked to the declining migration of *Mugil cephalus* (Linnaeus 1758) in the Northwestern Pacific [10]. Similarly, increasing SST has led to reduced diversity and stability in community structures along the northern coast of China [11], and has shifted fish community compositions towards more warm-affinity species along the western coast of Italy [12]. Furthermore, rising SST has the potential to perturb the microbial metabolism of coral, endangering the symbiotic relationship between corals and microbes, which results in reduced coral coverage and species diversity [13,14]. Coastal waters, critical for mariculture, experience significant SST fluctuations, especially in areas impacted by domestic and industrial effluents. For instance, a massive mortality event affected the Zhikong scallop (*Chlamys farreri* Jones & Preston 1904) populations in Shandong, China in 1997 [15] and the bay scallop (*Argopecten irradians* Lamarck 1819) fishery along the eastern coast of the United States collapsed during 2019–2021 due to habitat warming and hypoxia [16], highlighting the vulnerability of marine organisms to rising sea temperatures. Thus, rising sea temperatures pose a significant threat to marine organisms.

Marine organisms, primarily poikilotherms, exhibit various adaptations to heat stress, including changes in adaptive behavior [10,17], metabolic adjustments [18], and immune responses [19,20]. These adaptions often involve the synthesis of different biomacromolecules, which are regulated by specific gene expressions. One key player in this regulatory process is the Target of Rapamycin (TOR), first identified in *Saccharomyces cerevisiae* (Meyen 1883) [21], which is a highly conserved serine/threonine protein kinase that phosphorylates downstream effectors such as ribosomal protein S6 Kinase (S6K), eukaryotic translation initiation factor 4E-Binding Protein (4E-BP), and UNC-51-like autophagy activating Kinase 1 (ULK1), playing a central role in cell growth and metabolism [22,23]. TOR exists in two complexes, TOR complex 1 (TORC1) and TORC2. TORC1, widely existing in all eukaryotic organisms, is sensitive to rapamycin and significantly influences growth [24], reproduction [25,26], immunity [27,28], aging [29,30,31], and organismal responses to external changes [24,32,33,34]. Structurally, TORC1 comprises three core subunits: TOR, Regulatory-associated Protein of TOR (RAPTOR), and Lethal with SEC13 protein 8 (LST8), along with another two endogenous inhibitors, DEP-domain containing TOR-interacting protein (DEPTOR) and the Proline-rich protein kinase B (AKT) Substrate of 40 kDa (PRAS40). TORC1 integrates signals from nutrient levels, energetic demands, and environmental stresses to regulate cellular metabolism [33,35], such as modulating AMP-activated protein kinase (AMPK)-mediated energy budget [36,37], and influencing the synthesis of proteins, lipids, and autophagy processes by perceiving amino acids and some other growth factors [33]. Moreover, the level of TORC1 formation is correlated with overall mitochondrial activity and plays a significant role in regulating both oxygen consumption and oxidative capacity [38,39]. Despite considerable research on mammals and single-celled organisms like yeast, and some studies on nutrition and immunity in bivalve mollusks [40,41], investigations on scallops remain limited.

Scallops, a notable family of bivalves, hold significant economic importance in mariculture, and they easily suffer from oxidative damage caused by increases in temperature [42]. The bay scallop (*A. irradians*) is known for its rapid growth, contributing approximately 0.8 million tons annually and accounting for up to ~45% of China’s scallop production in recent years [43]. As a typically eurythermal warm water species, the bay scallop thrives in a wide temperature range from −1 °C to 31 °C, with optimal growth temperatures ranging from 18 °C to 28 °C [44]. However, prolonged heat stress has been shown to reduce survival and increase mortality rates among the bay scallops [45]. Consequently, this study focuses on the thermotolerance driven by the TORC1 to decipher their genetic regulations of bay scallops to elevated seawater temperatures. In this study, we conducted a genome-wide scan using bioinformatic methodologies to comprehensively identify TORC1-coding genes in *A. i. irradians* (Lamarck 1819). Structural and phylogenetic analyses of the *A. i. irradians* TORC1 (AiTORC1) subunits were then performed to elucidate the evolutionary relationships. Furthermore, the spatiotemporal expression of AiTORC1-coding genes in embryos/larvae at various developmental periods and healthy adult tissues were analyzed, providing insights into their role in development and tissue-specific functions. A subsequent heat stress experiment was carried out by exposing bay scallops to 32 °C to investigate the expression of AiTORC1-coding genes in temperature-sensitive tissues such as the mantle, gill, hemocyte, and heart [42] over diverse durations (0 h, 6 h, 12 h, 24 h, 3 d, 6 d, and 10 d). Finally, bay scallops were injected with rapamycin to assess changes in metabolic rate, cardiac performance under imitatively sustained warming conditions, and oxidative stress levels in different tissues 24 h post-treatment to explore the mechanisms through which TORC1 influences thermotolerance at both the individual and tissue levels. This comprehensive study sheds light on the pivotal roles of AiTORC1 in managing the physiological responses in bay scallops to temperature fluctuations, thereby offering valuable insights that could improve the sustainability and health of bay scallop aquaculture.

## 2. Materials and Methods

### 2.1. Database Mining, Gene Identification, and Sequence Analysis

Homologous protein sequences of TORC1 subunits from *Homo sapiens* (Linnaeus 1758), Mus musculus (Linnaeus 1758), *Xenopus laevis* (Daudin 1802), *Danio rerio* (Hamilton 1822), and *Crassostrea gigas* (Thunberg 1793) (Table S1) were retrieved from the NCBI (http://www.ncbi.nlm.nih.gov, accessed on 15 September 2022), Ensembl (http://asia.ensembl.org/index.html, accessed on 15 September 2022), and OysterBase (http:// oysterdb.com.cn/, accessed on 1 September 2024) database. These sequences were then mapped to the genome and transcriptome of *A*. *i*. *irradians* using the TBLASTN and BLASTP algorithms to identify the corresponding coding sequences (cDNAs) and AiTORC1 subunit protein sequences, respectively. Redundant isoforms that mapped to identical genomic locations were removed, leaving only unique and complete transcripts for subsequent analyses. The Simple Modular Architecture Research Tool (SMART, http://smart.embl.de/, accessed on 17 September 2022) was employed to recognize the conserved domains of each AiTORC1 subunit. Molecular weight and theoretical putative isoelectric points (pI) were predicted using the compute pI/Mw tool (http://web.expasy.org/compute_pi/, accessed on 17 September 2022). Furthermore, the prediction and annotation of secondary structures of each AiTORC1 subunit were conducted using PRABI (https://npsa-pbil.ibcp.fr/, accessed on 17 September 2022) and Geneious Prim 4.8.3 (http://www.geneious.com/, accessed on 17 September 2022), respectively, while the SWISS-MODEL (https://swissmodel.expasy.org/, accessed on 18 September 2022) was utilized to predict their tertiary structures.

### 2.2. Multiple Sequence Alignment and Phylogenetic Analysis

Protein sequence alignments of the TORC1 subunits were performed using the ClustalW2 program within MEGA 11 (https://www.megasoftware.net/, accessed on 18 September 2022). Amino acid sequences of the TORC1 subunits from *A. i. irradians* and orthologs from both vertebrates and invertebrates (Table S2), such as *H*. *sapiens*, *M*. *musculus*, *Ictidomys tridecemlineatus* (Mitchill 1821), *Vombatus ursinus* (Desmarest 1822), *Bos taurus* (Linnaeus 1758), *X*. *laevis*, *Xenopus tropicalis* (Fritzsch & Grafe 1994), *D*. *rerio*, *Stegostoma tigrinum* (Linnaeus 1758), *Saccoglossus kowalevskii* (Kowalevsky 1865), *Drosophila melanogaster* (Meigen 1830), *Acanthaster planci* (Lamarck 1816), *Strongylocentrotus purpuratus* (Stimpson 1857), *C*. *gigas*, *Crassostrea angulata* (Lamarck 1819), *Pecten maximus* (Linnaeus 1758), *P*. *yessoensis*, *Aplysia californica* (Cooper & Brusca 1964), and *Dendronephthya gigantean* (Kölliker 1880), were selected for this analysis, and the sequences were obtained from the NCBI database. A phylogenetic tree was then constructed using the neighbor-joining (NJ) method in MEGA 11, with the robustness of the tree assessed by 1500 bootstrapping replications. The final refinement of the phylogenetic tree was conducted using iTOL (https://itol.embl.de/, accessed on 18 September 2022).

### 2.3. Sample Collection and Heat Stress Experiment

The transcriptome data for analyzing the spatio-temporal expression of AiTORC1-coding genes were provided by our laboratory. Briefly, in February 2016, over 500 bay scallops were artificially hybridized at the hatchery of Laizhou Haiyi Marine Seeds Co., Ltd. (Yantai, China). Then, various developmental stages of scallops—zygotes, 2–8 cells, blastula, gastrula, trochophores, D-shaped larvae, umbo larvae, and juvenile scallops (N > 1000 for each stage to ensure consistency across biological replicates)—were systematically sampled and stored in RNAlater (Sigma-Aldrich, St. Louis, MO, USA) for subsequent RNA-seq analysis. In February 2019, tissues including the hepatopancreas, foot, mantle, gill, gonad, kidney, smooth muscle, striated muscle, hemocyte, and heart were dissected (*n* = 3, respectively) from three randomly selected healthy adult bay scallops and then immediately frozen in liquid nitrogen before being stored at −80 °C.

For the heat stress experiment, in March 2020, 9 month old healthy bay scallops (N > 500) were transferred from Laizhou (Yantai, China) to our laboratory (Ocean University of China, Qingdao, China), following standard procedures [46]. The scallops were cultured in filtered and aerated seawater at approximately 22 °C, within the bay scallops’ optimal growth temperature range of 18–28 °C. After a week of acclimatization, 160 bay scallops were randomly selected and exposed to heat stress by being placed in seawater heated to 32 °C, aligning with the previously determined Arrhenius break temperature (ABT) for bay scallops [47]. The reported temperature-sensitive tissue samples (mantle, gill, hemocyte, and heart) were collected from three randomly selected scallops at various time points (0 h, 6 h, 12 h, 24 h, 3 d, 6 d, and 10 d), and promptly frozen in liquid nitrogen for subsequent RNA-seq analysis. Throughout the experiment, the seawater salinity was maintained at 31.7 ± 0.13 and the pH at 8.09 ± 0.05. All experiments procedures were performed in compliance with the guidelines and regulations of our school and local government.

### 2.4. RNA Isolation and RNA-Seq Analysis

Total RNA was released in lysis buffer containing guanidine isothiocyanate, β-mercaptoethanol, and sodium citrate, and then extracted multiple times with phenol–chloroform and precipitated with isopropanol following the protocol described by Hu et al. [48]. After digesting with DNase I (TaKaRa, Shiga, Japan), high quality RNA was obtained though re-extraction. RNA-seq libraries were constructed using the VAHTS mRNA-seq v2 Library Prep kit (Vazyme, Nanjing, China). Subsequently, the libraries were sequenced on an Illumina HiSeq 2000 platform to generate 150-bp paired-end reads. RNA-seq data from embryos/larvae across 8 developmental stages (zygotes, 2–8 cells, blastula, gastrula, trochophores, D-shaped larvae, umbo larvae, and juvenile scallops), as well as from 10 adult tissue types (hepatopancreas, foot, mantle, gill, gonad, kidney, smooth muscle, striated muscle, hemocyte, and heart) were analyzed to determine the spatio-temporal expression of AiTORC1-coding genes. Additional RNA-seq data from samples exposed to heat stress at 32 °C for varying durations (0 h, 6 h, 12 h, 24 h, 3 d, 6 d, and 10 d) were utilized to explore the regulatory effects on AiTORC1-coding genes in bay scallops.

### 2.5. Functional Verification via Inhibition of AiTORC1

In February 2024, bay scallops (N > 100) from Haiyi Seeding Group Co. (Yantai, China) were transported to our laboratory (Qingdao, China) following the established procedures [44]. After a week of acclimatization, 45 bay scallops of similar sizes were randomly selected and divided into five groups (*n* = 9 for each group), including a control group with no treatment, a blank group injected with 40 µL 75% dimethyl sulfoxide (DMSO) in phosphate buffer saline (PBS 1×) [49], and three experimental groups treated with 40 µL of rapamycin (Macklin, Shanghai, China) dissolved in 75% DMSO at concentrations of 500 µmol/L (500 µM), 5 mmol/L (5 mM), and 50 mmol/L (50 mM), respectively. The injections were administered into the central region of the striated muscle at a depth of approximately 6.5 mm. The bay scallops were labelled on their shells and maintained at 22 °C in filtered and aerated seawater.

### 2.6. Metabolism Indicators Measurement and Cardiac Performance Detection

One day post-inhibition, three scallops from each group were randomly selected for assessment of their oxygen consumption rate (OR), ammonia excretion rate (NR), and cardiac performance parameters, including heart rate (HR), heart amplitude (HA), and rate-amplitude product (RAP). Each scallop was individually placed in a 2.5 L wide-mouth bottle filled with filtered seawater and sealed with caps. The concentrations of dissolved oxygen (DO, mg·L^−1^) and ammonium nitrogen (NH_4_^+^-N, mg·L^−1^) were measured at 0 h and 2 h using a Professional Plus multiparameter instrument (YSI, Yellow Springs, Ohio, USA) for DO and by hypo bromate oxidimetry [50] for NH_4_^+^-N. Subsequently, the soft tissues of the scallops were desiccated to a constant weight at 60 °C and then weighed. The other 15 scallops (*n* = 3 for each group) underwent cardiac performance monitoring using a non-invasive method improved by Xing et al. [47,51]. Briefly, optical sensors CNY-70 (Newshift, Lisbon, Portugal) were affixed with blue tack to the shell adjacent to their cardiocoelom to capture infrared signal variation. The signals were amplified by AMP-03 (Newshift, Lisbon, Portugal), then filtered and transmitted to a computer via the PowerLab 16/35 portable digital recording instrument (ADInstruments, Sydney, Australia). Using LabChart software (v8.1.3, ADInstruments, Dunedin, New Zealand), cardiac performances were monitored in real-time. The experimental setup included a gradual increase in seawater temperature at a rate of 0.2 °C/min from 22 °C to 36 °C, with a 5 minute stabilization period after every 2.0 °C increase [52], during which HR (beats per min, bpm), HA (voltage, V), and RAP (HR times HA, bpm·V) were recorded and calculated.

### 2.7. Oxidative Stress Indexes Measurement

One day after inhibition, another three scallops from each group were selected for an evaluation of their antioxidant abilities. Principal parameters indicating antioxidant capacity, including superoxide dismutase (SOD), catalase (CAT), and ROS, were measured in the mantle, gill, gonad, kidney, muscle, hepatopancreas, and heart of the sampled scallops (*n* = 3). The biochemical assays were measured using protocols provided by Nanjing Jiancheng Bioengineering Institute (http://www.njjcbio.com, accessed on 25 February 2024). The SOD activity was measured using the hydroxylamine method (Kit No. A001-1), CAT activity was assessed using the visible light method (Kit No. A007-1-1), and ROS levels were determined by the 2′,7′-dichlorodihydrofluorescein diacetate-based chemical fluorescence method (Kit No. E004).

### 2.8. Data Analysis

RNA-seq data generated from samples under normal and heat stress conditions were analyzed to examine the expression profiles of AiTORC1-coding genes across different developmental stages and in various tissues of healthy adult bay scallops, as well as their response to high temperature stress. The RNA-seq data were formalized and presented in the form of transcripts per kilobase of exon model per million mapped reads (TPM). TPM values from embryos/larvae at 8 developmental stages and from 10 adult tissue types were laterally homogenized. In the heat stress experiment, the TPM at 0 h served as the control, and fold change (FC) was calculated using the formula TPM_test_/TPM_0h_ to indicate the relative expression change at different time points. Gene expression patterns were visualized using heat maps generated with OmicStudio tools (https://www.omicstudio.cn/tool, accessed on 23 September 2022), with TPMs or FCs presented on a logarithmic base 2 scale. To facilitate the description of the results, zygotes and smooth muscle were used as reference controls for calculating FCs across different developmental stages and in adult tissues, respectively.

In TOR inhibitor rapamycin injection experiment, OR (mg·g^−1^·h^−1^), and NR (mg·g^−1^·h^−1^) of individuals were calculated as:

OR = [(DO_0_ − DO_t_) × V]/(W × t) (1)

NR = [(N_t_ − N_0_) × V]/(W × t) (2)

where DO_0_ and DO_t_ are dissolved oxygen concentrations, and N_0_ and N_t_ are ammonium nitrogen concentrations in seawater before and after the experiment, mg·L^−1^. V is the volume of seawater in wide-mouth bottles, L; W is dry weight of each scallop, g; and t is the duration of the experiment, h.

The ABT of bay scallop was determined by analyzing HR changes following the methodology described by Dahlhoff et al. [53]. A heat map of RAP was created after it was laterally homogenized using the OmicStudio tools. Significant differences in AiTORC1-coding genes expressions between the test groups and the smooth muscle were assessed using DESeq2, with significance set at *p* < 0.05 or |log_2_ FC| > 1, *n* = 3. Similarly, the expression differences in the heat stress experiment, as well as the comparisons of metabolism indicators OR and NR, along with oxidative stress markers SOD, CAT, and ROS, among five groups in the TOR inhibitor injection experiment, were determined using IBM SPSS Statistics 25.0 (https://www.ibm.com/spss, accessed on 2 March 2024). Statistical significance was determined through independent-sample *t*-Tests (*p* < 0.05, *n* = 3) and one-way Analysis of Variance (ANOVA, *p* < 0.05, *n* = 3), respectively.

## 3. Results

### 3.1. Sequence Identification and Analysis

**Figure 1:**
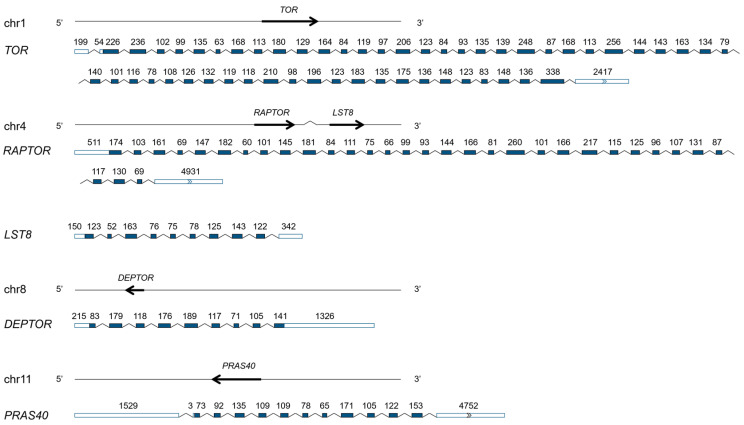
Genomic structure of AiTORC1-coding genes. Arrows indicate the gene locations on chromosomes. Exons within the ORF are shown as colored boxes, whereas 5′ and 3′ UTRs are represented by uncolored boxes, with introns represented by dashed lines. The number above each exon presents the exon length (bp).

Five AiTORC1-coding genes—*AiTOR* (*Arg0018340.1*, PP795900), *AiRAPTOR* (*Arg0077990.1*, PP795901), *AiLST8* (*Arg0078020.1*, PP795902), *AiDEPTOR* (*Arg0134570.1*, PP795903), and *AiPRAS40* (*Arg0186580.1*, PP795904)—were successfully identified in the genome and verified with transcriptome databases of bay scallops (Table 1 and Figure 1), as reported in other eukaryons, such as yeast and humans [54,55,56]. These genes encode the respective subunits AiTOR, AiRAPTOR, AiLST8, AiDEPTOR, and AiPRAS40, which together form the functional complex AiTORC1. The genes encoding *AiTOR*, *AiDEPTOR*, and *AiPRAS40* are located on chromosomes 1, 8, and 11, respectively, while *AiRAPTOR* and *AiLST8* are co-located on chromosome 4. The three core subunits coding genes—*AiTOR*, *AiRAPTOR*, and *AiLST8*—are 42,048, 30,308, and 25,971 bp, with open reading frames (ORF) lengths of 7500, 3963, and 957 bp, respectively. The genes encoding the endogenous inhibitors, *AiDEPTOR* and *AiPRAS40*, are 14,368 and 36,934 bp with ORF lengths of 1179 and 1212 bp, respectively. These genes contain 55, 33, 10, 9, and 13 exons and are predicted to encode AiTORC1 subunits comprising 2499, 1320, 318, 392, and 403 amino acids, respectively. Additional details, such as 5′ UTR length, 3′ UTR length, molecular weight, theoretical pI, and the number of α helices, β strands, coils, and turns are presented in Table 1.

**Figure 2:**
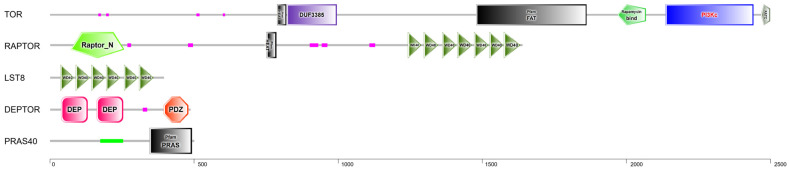
Structure and predicted protein domains of AiTORC1 by SMART analysis. Various domains are shown in boxes with different colors and shapes, with their names provided in boxes.

The functional domains of the AiTORC1 subunits, identified using the SMART database, are depicted in Figure 2. AiTOR features six domains, including HEAT repeats, the DUF3385 domain, the FAT domain, the Rapamycin bind domain (also known as FKBP-rapamycin binding, FRB domain), the Phosphoinositide 3-kinase catalytic (PI3Kc) domain, and the FATC domain, with the PI3Kc domain serving as the catalytic core. The FAT and FATC domains typically co-occur in the TOR (also known as the FK506-binding protein–rapamycin associated protein, FRAP), the ataxia-telangiectasia mutated (ATM) and transformation domain-associated protein (TRRAP) subfamilies of the Phosphoinositide-kinase superfamily. AiRAPTOR includes 10 domains, such as the Raptor N-terminal CASPase like (Raptor_N) domain, the HEAT domain and 7 repeated WD40 domains at the C terminus, which are crucial for protein–protein interactions. AiLST8 contains 6-tandem repetitive WD40 domains, enhancing AiTOR kinase activity and mediating its interaction with AiRAPTOR. AiDEPTOR featured two DEP domains and one PDZ domain, whereas AiPRAS40 harbored one Proline-rich AKT1 substrate 1 (PRAS) domain.

**Figure 3:**
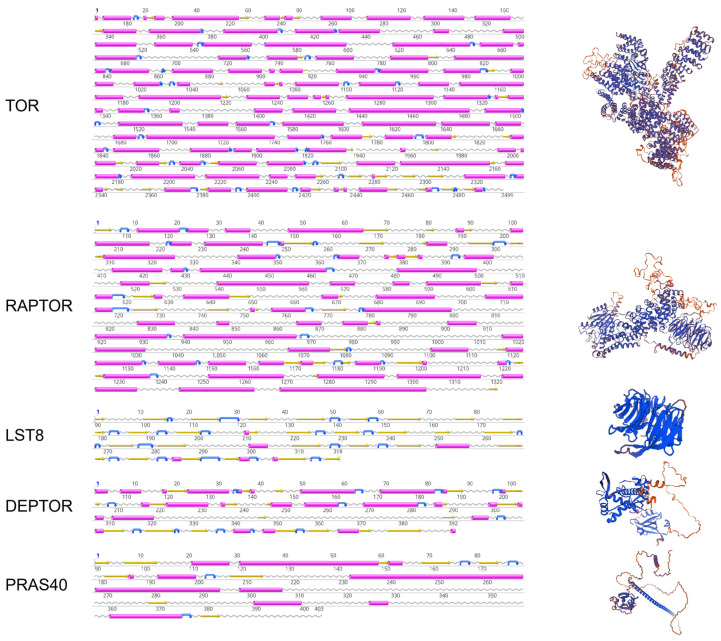
Secondary and tertiary structures of AiTORC1 subunits. Alpha helices are shown in pink cylinders, beta strands in yellow arrows, represent beta turns are in blue arrows, and random coils are in gray wavy lines.

**Figure 4:**
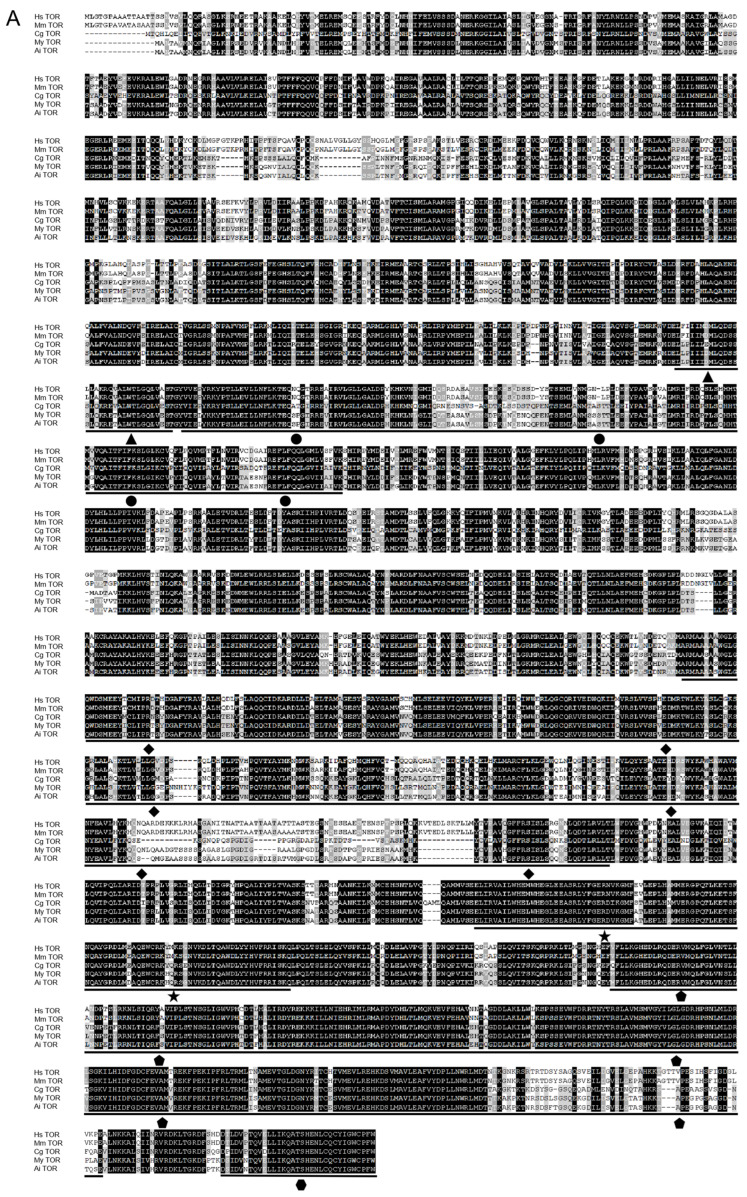

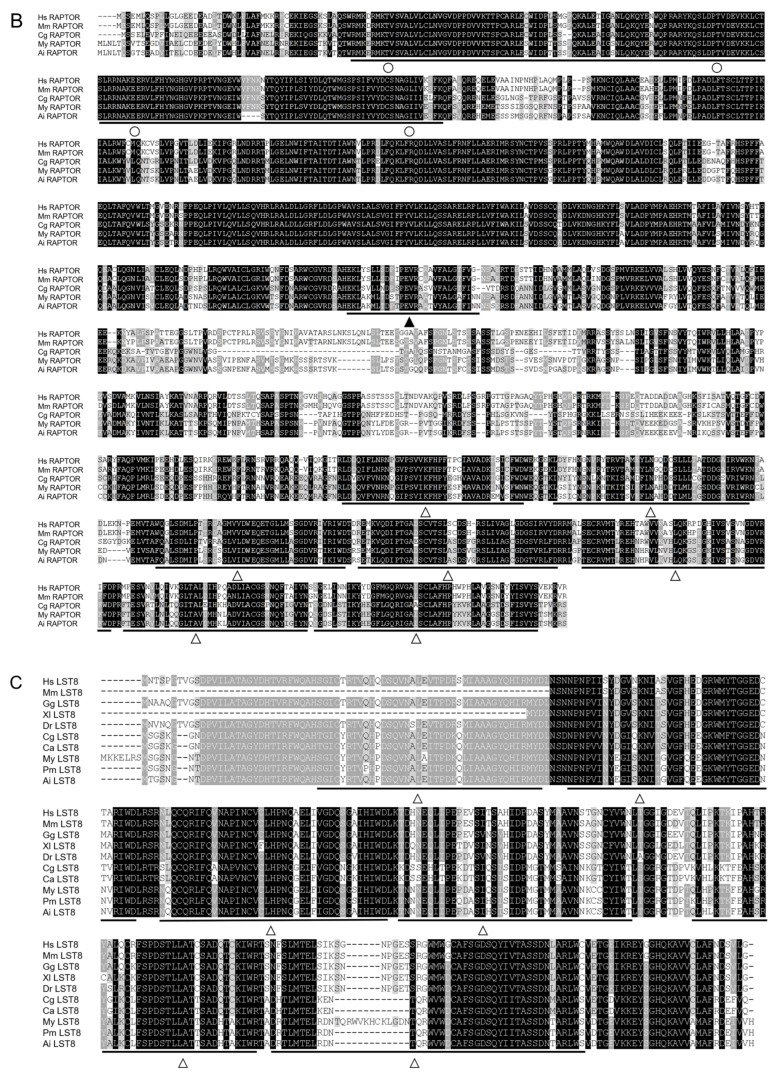

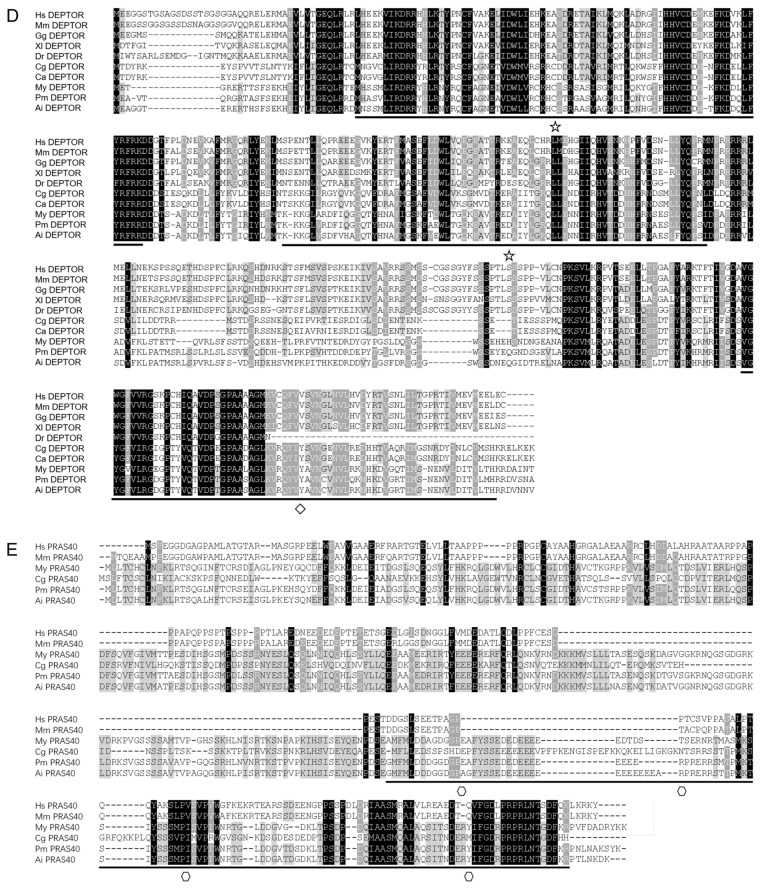
Multiple sequence alignment of AiTORC1 subunits, AiTOR (**A**), AiRAPTOR (**B**), AiLST8 (**C**), AiDEPTOR (**D**), and AiPRAS40 (**E**), amino acid sequences with homologous sequences from other species. Conserved amino acid are shaded in black. The gray-shaded regions represent similar amino acid residues. Sequences with low homology were not colored. Gaps are represented by dashes to improve the alignment. The symbols indicate various domains: The 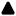 represent HEAT domains, the 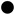 represents DUF3385 domain, the 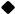 represents FAT domain, the 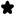 represents Rapamycin bind domain, the 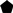 represents PI3Kc domain, the 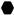 represents FATC domain, the 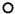 represents Raptor N domain, the 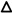 represent WD40 domains, the 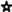 represent DEP domains, the 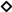 represents PDZ domain, and the 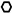 represents PRAS domain. Hs: *H*. *sapiens*, Mm: *M*. *musculus*, Gg: *G*. *gallus*, Xl: *X*. *laevis*, Dr: *D*. *rerio*, Ai: *A*. *i*. *irradians*, Pm: *P*. *maximus*, My: *M*. *yessoensis*, Cg: *C*. *gigas*, Ca: *C*. *angulata*. Accession numbers for these TORC1 subunits in other species are included in Supplementary Table S2.

Secondary structure predictions by PRABI suggest that, excluding AiLST8, all AiTORC1 subunits predominantly consist of α helices (Figure 3). This observation is supposed by their tertiary structures. A multiple sequence alignment of the TORC1 subunits (Figure 4), whose domains are marked with various symbols, shows extensive and high conservation across the selected eukaryons, particularly in the three core subunits compared to the two endogenous inhibitors.

### 3.2. Phylogenetic Analysis

**Figure 5:**
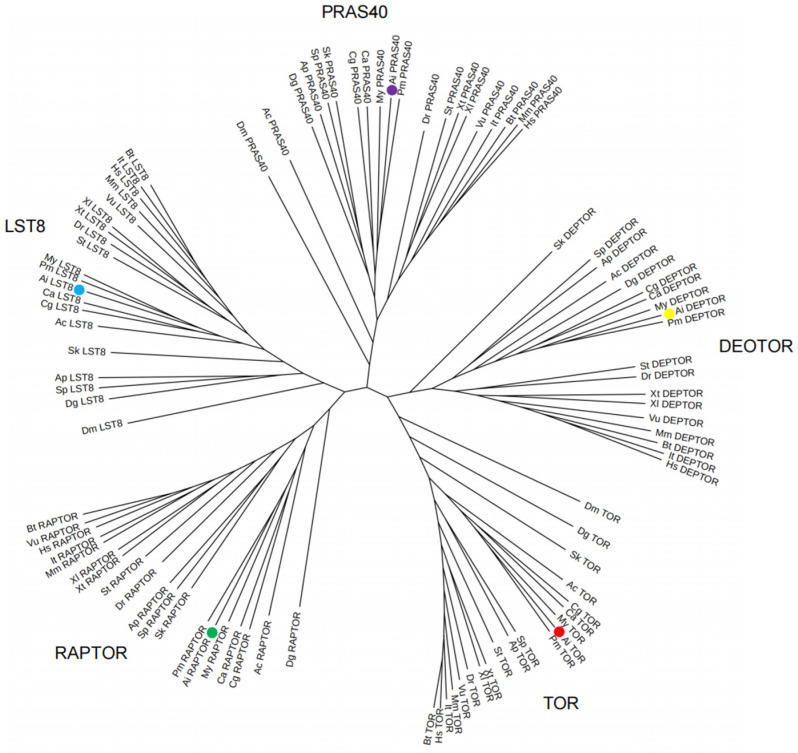
Phylogenetic tree of TORC1 based on the amino acid sequences of AiTORC1 subunits and homologous sequences from other species, constructed using the Neighbor-Joining method. AiTOR, AiRAPTOR, AiLST8, AiDEPTOR, and AiPRAS40 are represented by red, green, blue, yellow, and purple circles, respectively. Hs: *H*. *sapiens*, Mm: *M*. *musculus*, It: *I*. *tridecemlineatus*, Vu: *V*. *ursinus*, Bt: *B*. *taurus*, Xl: *X*. *laevis*, Xt: *X*. *tropicalis*, Dr: *D*. *rerio*, St: *S*. *tigrinum*, Sk: *S*. *kowalevskii*, Dm: *D*. *melanogaster*, Ap: *A*. *planci*, Sp: *S*. *purpuratus*, Cg: *C*. *gigas*, Ca: *C*. *angulata*, Pm: *P*. *maximus*, Py: *P*. *yessoensis*, Ai: *A*. *i*. *irradians*, Ac: *A*. *californica*, Dg: *D*. *gigantean*, The accession numbers of TORC1 subunits in other species are summarized in Supplementary Table S2.

The phylogenetic tree constructed using AiTORC1 and other TORC1 amino acid sequences from various vertebrates and invertebrates confirms the identities of TORC1 subunits in bay scallops (Figure 5). The NJ radiational tree displayed five distinct clusters corresponding to the TOR, RAPTOR, LST8, DEPTOR, and PRAS40 subfamilies. As anticipated, each TORC1 subunit was appropriately grouped into its respective clade. Within these clades, vertebrate sequences formed discrete sub-clades, while certain invertebrate subunits degraded into isolated sub-clades. Notably, sequences from mollusks, including *A. i. irradians*, *P. maximus*, *P. yessoensis*, *C*. *gigas,* and *C*. *angulata*, were clustered into independent clades, providing potential phylogenetic evidence for the classification of AiTORC1.

### 3.3. Spatio-Temporal Expression of AiTORC1-Coding Genes

**Figure 6:**
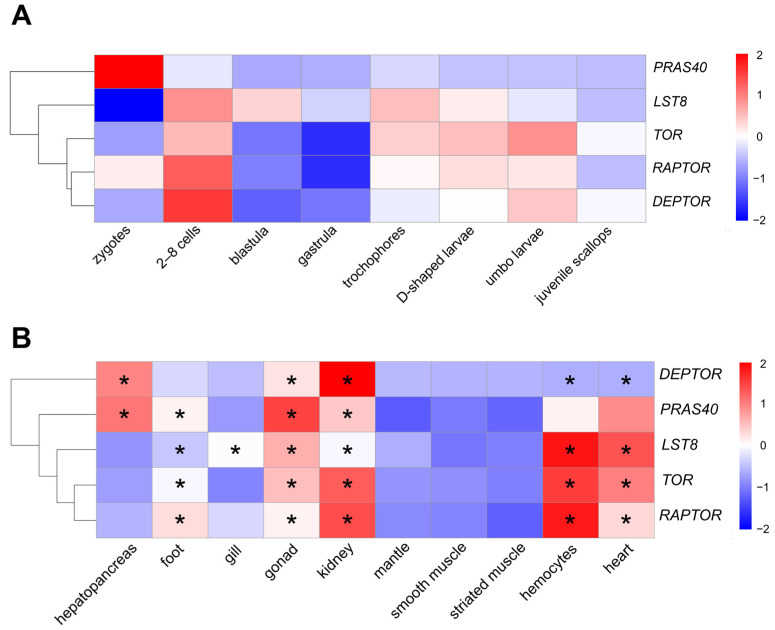
Spatio-temporal expression profiles of AiTORC1-coding genes. (**A**) The expression profiles of AiTORC1-coding genes from embryos or larvae across eight developmental stages (zygotes, 2–8 cells, blastula, gastrula, trochophores, D-shaped larvae, umbo larvae, and juvenile scallops) based on log_2_TPM after laterally homogenizing. (**B**) The expression profiles of AiTORC1 from 10 types of adult tissues (hepatopancreas, foot, mantle, gill, gonad, kidney, smooth muscle, striated muscle, hemocyte, and heart) based on log_2_TPM after laterally homogenizing. Significance (*p* < 0.05) is indicated through “*”.

As illustrated in Figure 6A, the five AiTORC1-coding genes displayed distinct expression patterns across all developmental stages. Notably, the three core subunits genes of AiTORC1, *AiTOR*, *AiRAPTOR,* and *AiLST8* peaked at the 2–8 cells stage, showing increases of 1.67 folds, 1.41 folds, and 2.86 folds, respectively, compared to the zygotes. Their expression decreased during the blastula and gastrula stages but surged again from the trochophores (1.58 folds, 0.97 folds, and 2.64 folds, respectively) to the umbo larvae stage (1.84 folds, 1.02 folds, and 2.19 folds, respectively). A similar pattern was observed in endogenous inhibitor AiDEPTOR-coding gene. Conversely, the other inhibitor coding gene, *AiPRAS40*, exhibited high expression in the zygotes but rapidly declined and remained low through to the juvenile scallops stage (0.08–0.24 folds), markedly differing from the other four subunits. TPMs of *AiLST8* and *AiPRAS40* were substantially higher than those for the other three subunits at each stage (Supplementary data).

Tissue-specific expression patterns of the five AiTORC1-coding genes in adult bay scallop tissues were also examined (Figure 6B). *AiTOR*, *AiRAPTOR*, and *AiLST8* showed significantly (*p* < 0.05) higher expression levels in the gonad, kidney, hemocyte, and heart samples, peaking in the tissue of hemocytes (3.93 folds, 3.79 folds, and 3.46 folds, respectively, compared to the corresponding expression in the smooth muscle). These three core subunits coding genes together expressed at lower levels in the hepatopancreas, mantle, smooth muscle, and striated muscle, and showed diverse expressions in the foot. The two endogenous inhibitors, *AiDEPTOR* and *AiPRAS40*, were significantly (*p* < 0.05) more expressed in the gonad (20.65 folds and 2.82 folds), kidney (73.12 folds and 2.07 folds), and hepatopancreas (37.95 folds and 2.53 folds). *AiPRAS40* also showed higher expression in the foot (1.83 folds, *p* < 0.05), hemocyte (1.84 folds) and heart (2.42 folds), whereas *AiDEPTOR* was not expressed in the hemocyte and was significant lower in the heart (0.14 folds, *p* < 0.05).

### 3.4. Regulations of AiTORC1-Coding Genes with Heat Stress

**Figure 7:**
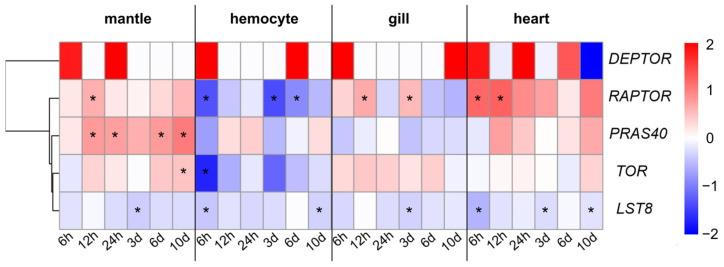
Expression regulations of AiTORC1-coding genes to high-temperature (32 °C) stimulation assessed in four types adult tissues (mantle, hemocyte, gill, and heart) and expressed as log2FC. Significance (*p* < 0.05) is indicated through “*”.

The expression profiles of AiTORC1-coding genes were determined in various temperature-sensitive tissues (the mantle, gill, hemocyte, and heart) subjected to a temperature of 32 °C at different time durations (0 h, 6 h, 12 h, 24 h, 3 d, 6 d, and 10 d) for an investigating of their regulatory responses to heat stress (Figure 7). Under these conditions, the expression patterns of *AiTOR*, *AiRAPTOR*, *AiLST8,* and *AiPRAS40* in the four tissues exhibited a generally synchronized fluctuation over time. However, *AiDEPTOR* was not expressed at the baseline temperature of 22 °C (0 h) in the mantle, hemocyte, and gill, and similar non-expression was also observed at various time points during the heat stress experiment (12 h, 3 d, 6 d, and 10 d in mantle, 12 h, 24 h, 3 d, and 10 d in hemocyte, 12 h, 24 h, 3 d, and 6 d in gill and 10 d in heart). The AiTORC1-coding genes (except for *AiLST8*) were generally up-regulated in the mantle, gill, and heart but were mainly down-regulated in the hemocyte, indicating a tissue-specific regulation pattern. Notably, two of the three core subunits, *AiTOR* and *AiRAPTOR*, exhibited similar regulatory trends across the examined tissues, with up-regulations in the mantle and heart, mixed regulations in the gill, and down-regulations in the hemocyte. Conversely, the core subunit-coding gene, *AiLST8*, was uniformly down-regulated across all four tissues at all time points (0.68–0.99 folds). The inhibitor AiPRAS40 coding gene also showed distinct tissue-specific expressions, being consistently up-regulated in the mantle and heart, akin to *AiTOR* and *AiRAPTOR*, but generally down-regulated in the hemocyte and gill. It is worth noting that *AiDEPTOR* displayed a prominent time-dependent regulation in all four tissues following heat stimulation, with expression noted at intermittent times such as 6 h and 24 h in the mantle, 6 h and 6 d in the hemocyte, and 6 h and 10 d in the gill, respectively, while in the heart, expression fluctuated between up- and down-regulation across different time points.

### 3.5. Effects of Inhibiting AiTORC1 on Metabolism and Thermotolerance

**Figure 8:**
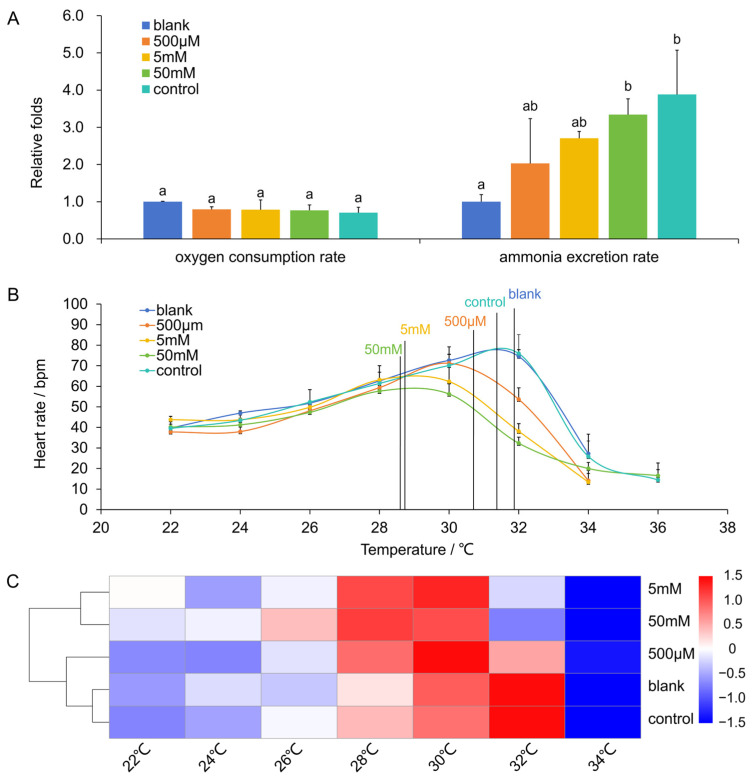
Effects of TOR inhibitor rapamycin on metabolism indicators in bay scallops following 24 h treatment. (**A**) Changes in oxygen consumption and ammonia excretion in bay scallops 24 h post treatment. Values that lack a single conserved letter differ significantly (*p* < 0.05) from each other. (**B**) Heart rate variations in bay scallops with temperature rising from 22 °C to 36 °C 24 h post treatment. (**C**) RAP variations in bay scallops with temperature from 22 °C to 34 °C 24 h post treatment.

Functional verification of AiTORC1’s role in metabolism was conducted in bay scallops treated with the TOR inhibitor rapamycin for 24 h, during which metabolic indicators such as OR, NR, HR, and RAP together showing metabolic intensity changes were observed (Figure 8). A slight decrease in OR was noted across the three experimental concentrations (500 µM, 5 mM, and 50 mM) compared to the control group. Notably, a considerable variation in NR was discovered among the five groups, with a significant reduction in the blank group compared to the control group (NR for the control was 3.88 folds higher than that of the blank), and increased with the rapamycin concentrations (2.03 folds, 2.71 folds, and 3.34 folds, respectively; the increase between 50 mM and blank was significant, *p* < 0.05), showing a positive correlation (Pearson r = −0.65, *p* = 0.02). This suggests that while rapamycin suppressed OR, it elevated NR in a dosage-dependent manner.

The inhibition of AiTORC1 further affected the scallop’s thermotolerance in terms of cardiac performance. As shown in Figure 8B, HR in the scallops increased similarly across all groups until reaching a peak, followed by a sharp decline when individuals from different groups suffered arrhythmia. There were no significant differences in resting HRs (RHR) among the groups (37.78−43.76 bpm at 22 °C). As the temperature of the seawater rose, the HRs of the scallops among the groups gradually increased, with no significant difference up to 28 °C (except at 24 °C, the difference between blank and 5 mM was significant, *p* < 0.05). However, scallops in the three experimental groups reached a maximum HR at significantly (*p* < 0.05) lower ABTs (30.34 ± 0.32 °C, 28.89 ± 0.33 °C and 28.55 ± 0.10 °C for 500 µM, 5 mM, and 50 mM groups, respectively), compared to the control (31.64 ± 0.71 °C) and blank (31.92 ± 0.25 °C) groups. Additionally, a positive correlation (Pearson r = −0.74, *p* = 0.50) was noted between the doses of the TOR inhibitor and the values of ABTs of the excremental groups. Furthermore, RAP was assessed to gauge the effect of AiTORC1 on cardiac energy metabolism in bay scallops (Figure 8C). RAPs patterns in scallops demonstrated a rise with increasing temperature, reaching peaks at 28 °C, 30 °C, or 32 °C, before experiencing a precipitous drop to notably low levels. Specifically, the RAPs of individuals in the experimental groups peaked earlier at 28 °C (205.26 bpm·V for 50 mM) or 30 °C (254.89 and 208.99 bpm·V for 500 µM and 5 mM, respectively), showing a tendency to reach the RAP extreme values in advance compared to those in the blank and control groups (221.93 and 243.34 bpm·V, respectively) at 32 °C. These observations strongly suggest that the inhibition of AiTORC1 can impair cardiac performance by disrupting energy metabolism, thereby reducing thermotolerance in bay scallops.

### 3.6. TORC1 Affecting Thermotolerance via Regulating Antioxidant Ability

**Figure 9:**
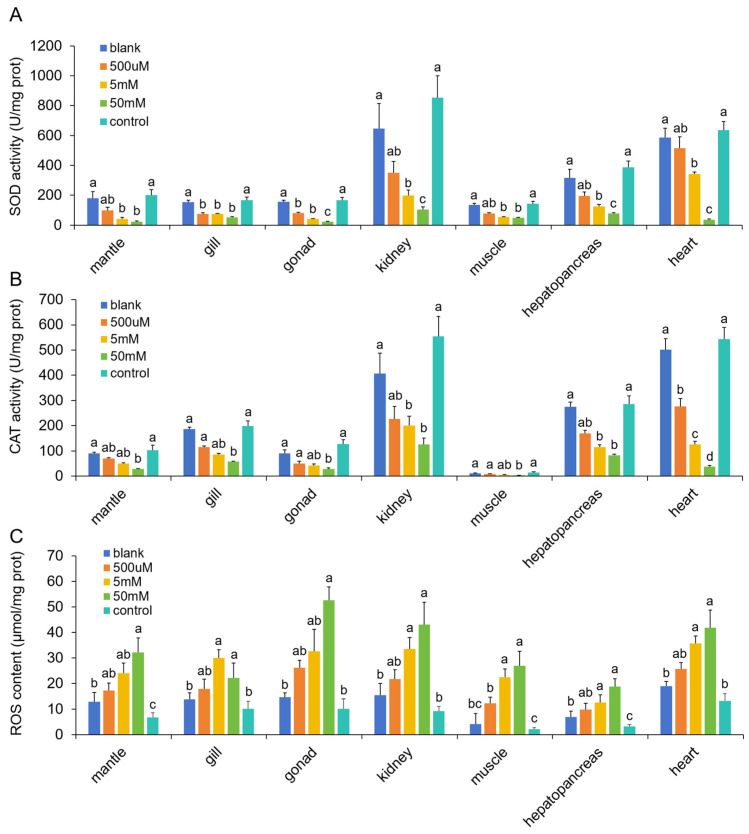
Effects of TOR inhibitor rapamycin on antioxidant ability assessment in bay scallops following 24 h treatment. (**A**) SOD activity. (**B**) CAT activity. (**C**) ROS content. Values that lack a single conserved letter differ significantly (*p* < 0.05) from each other.

To investigate the effects of AiTORC1 inhibition on antioxidant capacity, which plays a critical role in thermotolerance, we analyzed the functional status of key antioxidant markers, including SOD, CAT, and ROS. The activity patterns of SOD (Figure 9A) and CAT (Figure 9B) across tissues were similar, with a significant (*p* < 0.05) positive correlation between the two antioxidant enzymes in each tissue, with coefficients ranging from 0.91 to 0.99. Both SOD and CAT activities were significantly (*p* < 0.05) higher in the control and blank groups but showed a decreasing trend in the experimental groups (500 µM, 5 mM, and 50 mM), displaying a clear dose-dependent inhibition. In contrast, ROS levels exhibited an inverse relationship with SOD and CAT activities, with high negative correlation coefficients ranging from −0.796 to −0.995. Correspondingly, ROS levels were significantly (*p* < 0.05) higher in the experimental groups and increased with the dosage compared to the control and blank groups (Figure 9C). Interestingly, the heart tissue showed relatively higher levels of SOD, CAT, and ROS, which were closely related to metabolism and thermotolerance indicators. In detail, Pearson correlation coefficients between SOD/CAT/ROS and NR were 0.87 (*p* = 0.13), 0.99 (*p* = 0.01), and −0.98 (*p* = 0.02), respectively. Similarly, the correlations between SOD/CAT/ROS and ABT were −0.923 (*p* = 0.08), −0.99 (*p* = 0.01), and 0.987 (*p* = 0.01). These data reveal that AiTORC1 inhibition reduces the bay scallop’s antioxidant capacity, disrupts energy metabolism in the heart, and ultimately impairs cardiac performance, leading to decreased thermotolerance.

## 4. Discussion

TORC1 regulates growth and metabolism by controlling anabolic and catabolic processes in response to various signals [24,26]. Despite its importance, there is a notable lack of comprehensive research on the full spectrum of TORC1-coding genes in bivalves. In this study, we conducted a comprehensive examination of the genome and transcriptome of a marine bivalve bay scallop, and successfully identified five TORC1-coding genes. Structural and phylogenetic analyses of TORC1 subunits were further conducted. We specifically assessed the spatio-temporal expression of AiTORC1-coding genes in embryos/larvae across 8 developmental stages and in 10 different kinds adult tissues. Furthermore, the expression regulations of AiTORC1-coding genes to heat stress at 32 °C were examined in four temperature-sensitive tissues of bay scallops across various time durations. Finally, we validated the functional impact of AiTORC1 on metabolism in bay scallops 24 h post-treatment with the TOR inhibitor rapamycin. Through genome-wide identification, structural and evolutional analysis, expression regulations, and functional verification, the present investigation thoroughly explored the molecular modulation of AiTORC1 in bay scallops, enhancing our understanding of its biological roles.

All five AiTORC1-coding genes were successfully identified in the bay scallops (*A*. *i*. *irradians*). Compared to the genes encoding two endogenous inhibitors, *AiTOR* and *AiRAPTOR* exhibited longer gene and ORF lengths, resulting in more complex proteins with larger molecular weights and intricate structures, which underpin their roles as crucial catalytic and scaffold subunits [57,58,59,60]. SMART predictions highlighted the evolutionary conservation of domains within the five TORC1 subunits, pinpointing several key conserved domains with catalytic or linking functions. AiTOR, like other TOR proteins from vertebrates and invertebrates, contains six different domains [58], with the FAT and FATC domains uniquely co-occurring and folding together to ensure the functionality of the PI3Kc domain [60]. The rapamycin-binding domain features a conserved site crucial for rapamycin to inhibit the TORC1 activity [61,62]. AiRAPTOR’s domain composition mirrors that reported in humans [63], with a notable abundance of the WD40 domain compared to other eukaryotes. AiLST8 also contains seven WD repeats and is tightly associated with the kinase domain of TOR [61]. The highly conserved structural features of the three core subunits across vertebrates and invertebrates, along with the expanded functional domains in a key subunit, likely ensure the robust functionality of the essential TORC1 core in the bay scallop. Moreover, the domains of the two inhibitors exhibited high consistency yet low sequence homology, potentially reflecting an adaptation to the fluctuating marine environment. The distinct clades of mollusks TORC1 subunits in the NJ phylogenetic tree, diverging from the vertebrate cluster, indicated a conserved yet distinct mechanism of the complex, as reported in yeasts and mammals [64]. In summary, evidence of AiTORC1-coding genes identification, domain prediction, and evolutionary analysis was presented, shedding light on the structural conservation and functional allocation of AiTORC1 in bay scallops.

The spatio-temporal expression of AiTORC1-coding genes revealed patterns indicative of developmental stage and tissue specificities. The expression of these genes across eight developmental stages indicated their pivotal role in the embryonic and larval development of bay scallops [65]. In zygotes, AiPRAS40 was the only subunit with a high level of gene expression, hinting that it has a role distinct from previously identified functions. Its elevated expression in the gonad (Figure 6B) may indicate the presence of maternal mRNA during the zygotic phase. The genes coding three core subunits (*AiTOR*, *AiRAPTOR*, and *AiLST8*), along with *AiDEPTOR*, showed their highest expressions during the 2−8 cells stage, underscoring their importance in DNA replication and protein synthesis, which are crucial for embryonic cleavage and blastocyst development, aligning with findings in mice [66,67]. Interestingly, the high expressions of *AiDEPTOR* at the 2−8 cells stage might contribute to promoting pluripotency and self-renewal, as reported in human embryonic stem cells [68]. Curiously, the reduced expressions of all five subunit-coding genes during the blastula and gastrula stages presents a novel finding that warrants further exploration. Furthermore, the elevated levels of AiTORC1 core subunits from the trochophores to the umbo larvae implied their involvement in larval growth and organ size regulation, potentially through the downstream transcription factor Forkhead box O (FoxO) [69,70]. As larvae progress to the swimming and feeding trochophore stage, the increased energy expenditure and synthesis of digestive enzymes likely necessitate the up-regulation of the core subunit-coding genes of AiTORC1 [71,72]. Moreover, the synchronous expression of *AiDEPTOR* with the core subunit-coding genes implied its role in apoptosis during larval metamorphosis [73]. In conclusion, AiTORC1 appears to play diverse functions in the embryonic and larval development of the bay scallop, exhibiting a stage-specific expression pattern.

The expressions of the five AiTORC1-coding genes in adult bay scallop tissues revealed significant differences among these tissues, with notably higher expression in the gonad and kidney. The 9 month old bay scallops used in this study were at the brink of sexual maturity, undergoing intense cell division and differentiation in the gonads, necessitating robust transcriptional and translational activities [74]. The pronounced expression of *PRAS40* in the gonads may reflect a unique role in TORC1 activation through insulin signaling pathways, paralleling findings in *D. melanogaster* [75]. The kidney, a highly metabolic tissue in marine mollusks [76], engages in signal transduction, biosynthesis of secondary metabolites, transport and catabolism, energy production and conversion, and amino acid transport and metabolism [77]. Thus, excess byproduct ROS might also damage the macromolecules inside kidney cells, thereby inhibiting metabolism and activating autophagy [78]. It is also the site of immune-related enzymes, such as superoxide dismutase, catalase, and myeloperoxidase synthesis [79], potentially under TORC1 regulation. Autophagy, crucial in the kidney, eradicates damaged organelles and macromolecules [80]. In humans, the inhibitor irisin has been shown to restore autophagy in kidneys by counteracting abnormal activation of the PI3K/AKT/mTOR signaling pathway [81]. The high expression of *AiDEPTOR* in the scallop kidney may indicate its role in autophagy to boost immune activity. In contrast, the hepatopancreas, the primary digestive tissue in shellfish, showed a different expression pattern of AiTORC1-coding genes compared to its regulatory role in hepatic metabolism observed in rainbow trout [82]. Given its function as an immune tissue where bacteria accumulate [83,84], the elevated expression of the two endogenous inhibitors-coding genes may be associated with combating pathogens by enhancing lysosome biogenesis, inducing autophagy, and supporting bacterial clearance within phagosomes through TORC1 kinase activity inhibition [85]. In bivalves, hemocyte, consisting of granulocytes, semigranulocytes and hyalinocytes, are crucial for nutrient transportation and immune defense [86]. The high expression of AiTORC1-coding genes in hemocyte may be linked to the synthesis of transport proteins and hydrolytic enzymes, given their phagocytic activity and enzyme content [87]. The heart, with its high energy requirements for sustained contraction, has low ATP reserves and a rapid rate of ATP hydrolysis [88]. Previous studies have suggested that TORC1 regulates mitochondrial activity by controlling the translation of mitochondrial-associated mRNAs, affecting cellular energy metabolism [89]. The elevated expression of AiTORC1 core subunits in the heart implies their importance in energy metabolism regulation. Conversely, the lower expression of AiTORC1-coding genes in the mantle and smooth/striated muscle might be attributed to a relatively inactive metabolism state during sampling. In summary, AiTORC1 is integral to the growth and various physiological functions of the bay scallop.

Heat stress can induce damage to intracellular proteins, DNA, lipids, and other important biomacromolecules directly or indirectly by causing oxidative stress [90], thereby impacting various physiological processes in organisms, including energy metabolism, immune response [91,92], as well as growth and apoptosis [93], a process in which TORC1 plays crucial regulatory roles. Heat stimulation leads to damage to essential biomacromolecules in cells, with DEPTOR capable of inhibiting TOR kinases activity [73], subsequently activating autophagy by phosphorylating ULK1 to degrade these damaged biomacromolecules. This likely underlies the rapid and robust expression of *AiDEPTOR* observed in four tissues under heat stress. Following an accumulation of damaged biomacromolecules over time, there arises a time-dependent necessity for AiDEPTOR in autophagy induction, as evidenced by observations at 24 h in the mantle, 6 d in the hemocyte, 10 d in the gill, 24 h, and 6 d in the heart, respectively (Figure 7). Notably, the synchronized expressions of the four subunit-coding genes (*AiRAPTOR*, *AiPRAS40*, *AiTOR,* and *AiLST8*) over the duration of the heat stress challenge suggest their interrelation via joint regulation, driving bay scallops to respond to heat stress. The mantle serves as the primary barrier against elevated temperatures [42], wherein up-regulations of *AiPRAS40*, *AiTOR,* and *AiRAPTOR* occurred to varying degrees. As observed in Figure 7, increased *AiTOR* and *AiRAPTOR* transcripts may indicate an active status of metabolism, and a high level of byproduct ROS, which can cause damage to macromolecules. Simultaneously, oxidative stress-induced damage caused by seawater warming necessitates AiPRAS40 to competitively bind to AiRAPTOR and inhibit AiTORC1 activity [94,95] in response to heat stress, potentially reducing intracellular metabolic levels and inducing growth arrest. Hemocyte represent the main cellular defense against heat stress, and the low expression levels of *AiTOR* and *AiRAPTOR* suggested weakened intracellular metabolic activities in hemocyte under heat stress [96]. Under high temperature stress, notable modifications in the morphological structures of the gill occurred, with reductions in gill flap thickness, gill filament width, and distance between filaments [97]. In scallops, the gill serves the dual role of both breathing and feeding, with increased expressions of *AiTOR* and *AiRAPTOR,* suggesting enhanced metabolism, presumably mitigating the effects of morphological alterations on the breathing and feeding functions of scallops. Previous research has verified increased energy metabolism in scallop gills under high temperature stress, potentially accounting for AiTORC1 activity [98]. The scallop heart serves as the central source of circulatory energy for the entire organism. Previous studies have shown that as temperatures rise, the HR of the bay scallop accelerated until its ABT (32.20 ± 0.25 °C), indicating an increased demand for energy support [47]. The tremendous up-regulation of *AiRAPTOR* in the heart could respond to energy signals mediated by AMPK [37] to ensure adequate energy supply for heart function maintenance. In summary, the four tissues exhibited distinct regulatory patterns in response to heat stimulation. It is evident that LST8 does not play a direct role in coping with heat stress, while the other four subunits of AiTORC1 regulate this process to varying degrees. And it is also interesting that, in *Arabidopsis thaliana* (Linnaeus 1753), Glc-mediated thermotolerance involved the induction of heat shock proteins (HSPs) via the TOR-E2 promoter binding factor α (E2Fα) signaling module [99], suggesting a potential role for TOR in influencing the activation and induction of HSPs, crucial in the scallop’s response to heat stress [100]. This may represent another regulatory mechanism of TORC1 in the bay scallop to resist thermal stimulation, warranting further investigation.

TORC1 serves as a pivotal hub for integrating signals from nutrients, energy status, and oxidative stress to regulate biological growth and metabolism [33,35,39]. Rapamycin, a potent TOR inhibitor widely used in investigations spanning from yeast to mammals, including various mariculture species, effectively suppresses AiTORC1 [101,102,103,104]. Inhibition of TOR by rapamycin-induced changes in metabolic indices of bay scallops, manifested as a slight decrease in OR and a significant increase in NR, indicated a successful suppression of AiTORC1. Particularly noteworthy is the elevated NR accompanying TOR inhibition, signifying a heightened nitrogen metabolic rate [105,106,107,108]. A similar finding was reported in *Siniperca chuatsi* (Basilewsky 1855) where TORC1 activation reduced the ammonia-N excretion [109], which together with our study collectively suggests that TORC1 plays a crucial regulatory role in N-metabolism. Alternatively, rapamycin-induced TORC1 inhibition potentially activated autophagy, evidenced by the NR increase, suggesting that micromolecules (especially amino acids) produced by rapamycin-treated individuals were not reused by cells [21,80]. It is conjectured that rapamycin effectively inhibits protein synthesis and induces autophagy, generating more amino acids for energy metabolism, as evidenced in mice [110]. Moreover, the enhanced ROS levels (Figure 9C) might be caused by the elevated NR (Figure 8A). As reported in mice, dysregulated amino acid metabolism contributed to oxidative stress [111]. These elevated ROS levels may further impair the SOD and CAT activity due to the consumption of them [42]. Interestingly, among all tissues, the closest correlations (Pearson r were 0.87, 0.99, and −0.98, respectively) between SOD/CAT/ROS and NR were discovered in the heart, and our previous research also noted a close correlation between the cardiac performance indicators and antioxidant markers in the bay scallop [112]. As a temperature-sensitive tissue vital for the circulation of scallops, the heart of the bay scallop has been extensively researched. [51,112]. Therefore, we focused on the effects of rapamycin on AiTORC1 inhibition subsequently affect individuals’ cardiac performance, indicating a resistance and adaptation to environmental stress, particularly thermotolerance [51]. As anticipated, AiTORC1-suppressed scallops decreased OR, accompanied by significantly reduced ABT values, potentially leading to oxygen deprivation in the heart and a shift from aerobic metabolism to partially anaerobic metabolism [113]. Scallops experience reduced fitness as water temperatures approach their ABT, which trigger antioxidant defenses [114]. In this study, a significant relationship between ABT and antioxidant markers in the heart was observed after TORC1 inhibition. TORC1 is crucial for the cellular response to oxidative stress [89,115], thus suppression of AiTORC1 caused to such non-lethal impacts appear earlier in bay scallops. Speculatively, AiTORC1 inhibition appears to impact the aerobic metabolism in scallop heart, decreasing thermotolerance. Similarly, changes in the trend of RAP, an indicator of the heart’s energy output and for oxygen supply demand [51], might also signify this metabolic shift and diminished energy metabolism post-rapamycin treatment. These significant metabolic changes suggested a close association between AiTORC1 and respiratory metabolism supplying energy for metabolic processes [116]. Comparable phenomena have also been reported in mammals, wherein the mTOR pathway significantly regulates basal oxygen consumption and oxidative capacity, with rapamycin treatment lowering oxygen consumption and ATP synthetic capacity [39]. ATP production capacity, crucial for energy metabolism, can be increased by stimulating the translation of mitochondria-related mRNAs, a process controlled by TORC1 [117]. Besides, DMSO has some antioxidant capacity [118], but more importantly, when DMSO reaches a certain concentration, it disrupts the intracellular redox balance and destabilizes proteins [119], leading to a decrease in the activity of SOD and CAT, and causing mild oxidative stress in the blank group. In summary, AiTORC1 suppression affected the energy metabolism and antioxidant capacity of bay scallops, and reduced their thermotolerance, highlighting the crucial role of AiTORC1 in helping bay scallops withstand external environmental warming. In addition to the heat stress caused by summer heatwaves, the effects of AiTORC1 on scallop juveniles transferred to open sea areas during the cooler spring months and adults at lower temperatures during the overwintering period should not be underestimated [120,121,122]. Moreover, our future studies will focus on comparisons of both subspecies *A. i. irradians* and *A. i. concentricus* (Say 1822), that will be helpful for a better understanding of heat and cold tolerance, and for gaining more insights into the evolutionary relationship of both subspecies, habitat segregation, and possible speciation processes.

## 5. Conclusions

In conclusion, we performed a comprehensive genome-wide identification and analysis of TORC1-coding genes in *A. i. irradians*. Five AiTORC1-coding genes were identified, namely *AiTOR*, *AiRAPTOR*, *AiLST8*, *AiDEPTOR,* and *AiPRAS40* from the genomic and transcriptomic data of bay scallops. Their conservation and evolutionary relationships were established through multiple sequence alignments and phylogenetic analyses. The spatio-temporal expression patterns reflected AiTORC1’s functional roles across various developmental stages of embryos/larvae and in adult tissues. Regulations of AiTORC1-coding genes exhibited distinct tissue specify and temporal dependency in bay scallops when challenged with high temperatures. Functional verification by inhibiting AiTORC1 revealed enhanced catabolic activity, increased oxidative stress, and a significant reduction in thermotolerance in *A. i. irradians*. Further investigations could provide deeper insights into the distinct functional allocation of TORC1 across different tissues in scallops.

## Figures and Tables

**Table 1:** Sequence features of AiTORC1.

Genes	*AiTOR*	*AiRAPTOR*	*AiLST8*	*AiDEPTOR*	*AiPRAS40*
Chromosome	1	4	4	8	11
Total length (bp)	42,048	30,308	25,971	14,368	36,934
5′UTR length (bp)	253	511	150	215	1532
3′UTR length (bp)	2417	4931	342	1326	4752
ORF length (bp)	7500	3963	957	1179	1212
Number of amino acids	2499	1320	318	392	403
Molecular weight (kDa)	282.88	147.21	35.67	45.25	45.30
Theoretical pI	6.99	6.35	5.92	7.33	5.06
Number of exons	55	33	10	9	13
Number of introns	54	32	9	8	12
Number of α helices	103	61	6	26	10
Number of β strands	41	30	28	19	9
Numbers of coils	119	67	41	31	19
Number of turns	41	24	18	13	4

## Data Availability

The sequences of the five AiPC4s have been submitted to GenBank (accession number PP795900–PP795904). The original contributions presented in the study are included in the article/Supplementary Material. Further inquiries can be directed to the corresponding author.

## Supplementary Materials

The following supporting information can be downloaded at: https://www.mdpi.com/article/10.3390/antiox13111359/s1, Table S1: Accession numbers of TORC1 complex subunits homologous sequences for BLAST; Table S2: Accession numbers of TORC1 complex subunits homologous sequences for phylogenetic analysis; Data S1: Expression levels (TPM) of AiTORC1-coding genes in embryos/larvae at all developmental stages, in healthy adult tissues, and in four tissues being processed under 32 °C stress with different time durations.
