# Supplementary material for: TORC1 Regulates Thermotolerance via Modulating Metabolic Rate and Antioxidant Capacity in Scallop Argopecten irradians irradians
Source: Antioxidants (Basel). 2024 Nov 6;13(11):1359. doi: 10.3390/antiox13111359 (PMC11591371; doi:10.3390/antiox13111359)
Supplement: Supplementary file 1 [file antioxidants-13-01359-s001.zip › Supplementary Table.pdf]

Table S1. Accession numbers of TORC1 complex subunits homologous sequences for BLAST.

Species	Proteins	Accession numbers
Homo sapiens	TOR	NP_001373429.1
	RAPTOR	NP_065812.1
	LST8	NP_001186102.1
	DEPTOR	NP_073620.2
	PRAS40	NP_115751.3
Mus musculus	TOR	NP_064393.2
	RAPTOR	NP_083174.2
	LST8	NP_001239392.1
	DEPTOR	NP_663445.2
	PRAS40	NP_001277623.1
Xenopus laevis	TOR	XP_018081147.1
	RAPTOR	XP_018090569.1
	LST8	NP_001083382.1
	DEPTOR	XP_018123721.1
	PRAS40	NP_001088244.1
Danio rerio	TOR	NP_001070679.3
	RAPTOR	XP_005157410.1
	LST8	NP_001307317.1
	DEPTOR	XP_005173580.1
	PRAS40	XP_692511.2
Crassostrea gigas	TOR	XP_011432165.2
	RAPTOR	XP_034311661.1
	LST8	XP_011441680.2
	DEPTOR	XP_011437241.2
	PRAS40	XP_011424050.2

Table S2. Accession numbers of TORC1 complex subunits homologous sequences for phylogenetic analysis.

Species	Proteins	Accession numbers
Homo sapiens	TOR	NP_001373429.1
	RAPTOR	NP_065812.1
	LST8	NP_001186102.1
	DEPTOR	NP_073620.2
	PRAS40	NP_115751.3
Mus musculus	TOR	NP_064393.2
	RAPTOR	NP_083174.2
	LST8	NP_001239392.1
	DEPTOR	NP_663445.2
	PRAS40	NP_001277623.1
Ictidomys tridecemlineatus	TOR	XP_005317508.1
	RAPTOR	XP_005332694.1
	LST8	XP_021590604.1
	DEPTOR	XP_005316257.1
	PRAS40	XP_005336764.1
Vombatus ursinus	TOR	XP_027699623.1
	RAPTOR	XP_027705944.1
	LST8	XP_027718744.1
	DEPTOR	XP_027709828.1
	PRAS40	XP_027715119.1
Bos taurus	TOR	XP_002694089.2
	RAPTOR	NP_001179059.3
	LST8	NP_001030488.1
	DEPTOR	NP_001095782.1
	PRAS40	NP_001076903.1
Xenopus laevis	TOR	XP_018081147.1
	RAPTOR	XP_018090569.1
	LST8	NP_001083382.1
	DEPTOR	XP_018123721.1
	PRAS40	NP_001088244.1
Xenopus tropicalis	TOR	XP_031761370.1
	RAPTOR	NP_001123843.1
	LST8	NP_001011443.1
	DEPTOR	XP_004915116.1
	PRAS40	NP_001011102.1
Danio rerio	TOR	NP_001070679.3
	RAPTOR	XP_005157410.1
	LST8	NP_001307317.1

	DEPTOR	XP_005173580.1
	PRAS40	XP_692511.2
Stegostoma tigrinum	TOR	XP_048417158.1
	RAPTOR	XP_048410853.1
	LST8	XP_048408098.1
	DEPTOR	XP_048384358.1
	PRAS40	XP_048377137.1
Saccoglossus kowalevskii	TOR	XP_002739087.1
	RAPTOR	XP_006824701.1
	LST8	XP_002736782.1
	DEPTOR	XP_006819350.1
	PRAS40	XP_006818294.1
Drosophila melanogaster	TOR	NP_001260427.1
	LST8	NP_572572.1
	PRAS40	NP_001303349.1
Acanthaster planci	TOR	XP_022096916.1
	RAPTOR	XP_022092317.1
	LST8	XP_022092405.1
	DEPTOR	XP_022088199.1
	PRAS40	XP_022083715.1
Strongylocentrotus purpuratus	TOR	XP_030842943.1
	RAPTOR	XP_030840517.1
	LST8	XP_030840258.1
	DEPTOR	XP_030839118.1
	PRAS40	XP_030829365.1
Crassostrea gigas	TOR	XP_011432165.2
	RAPTOR	XP_034311661.1
	LST8	XP_011441680.2
	DEPTOR	XP_011437241.2
	PRAS40	XP_011424050.2
Crassostrea angulata	TOR	XP_052715346.1
	RAPTOR	XP_052688008.1
	LST8	XP_052688441.1
	DEPTOR	XP_052687141.1
	PRAS40	XP_052685893.1
Pecten maximus	TOR	XP_033724616.1
	RAPTOR	XP_033755950.1
	LST8	XP_033756002.1
	DEPTOR	XP_033736714.1
	PRAS40	XP_033752548.1
Patinopecten yessoensis	TOR	XP_021350104.1

	RAPTOR	XP_021350516.1
	LST8	XP_021350510.1
	DEPTOR	XP_021378961.1
	PRAS40	XP_021351376.1
Aplysia californica	TOR	XP_035824599.1
	RAPTOR	XP_035826281.1
	LST8	XP_012936932.1
	DEPTOR	XP_012944000.1
	PRAS40	XP_012942381.1
Dendronephthya gigantea	TOR	XP_028391632.1
	RAPTOR	XP_028395164.1
	LST8	XP_028395489.1
	DEPTOR	XP_028414602.1
	PRAS40	XP_028411100.1
